# Understanding Factors Leading to Primary Cesarean Section and Vaginal Birth After Cesarean Delivery in the Friuli-Venezia Giulia Region (North-Eastern Italy), 2005–2015

**DOI:** 10.1038/s41598-019-57037-y

**Published:** 2020-01-15

**Authors:** L. Cegolon, G. Mastrangelo, G. Maso, G. Dal Pozzo, L. Ronfani, A. Cegolon, W. C. Heymann, F. Barbone

**Affiliations:** 1 Institute for Maternal & Child Health, IRCCS “Burlo Garofolo”, Trieste, Italy; 2 Local Health Unit N.2 “Marca Trevigiana”, Public Health Department, Treviso, Italy; 3 Padua University, Department of Cardio-Thoracic & Vascular Sciences, Padua, Italy; 4 Hospital “Villa Salus”, Obstetric & Gynecology Unit, Venice, Italy; 5 University of Macerata, Department of Political Sciences, Comunication and International Relationships, Macerata, Italy; 6 Florida Department of Health, Sarasota County Health Department, Sarasota, Florida USA; 7 Florida State University, College of Medicine, Department of Clinical Sciences, Sarasota, Florida USA

**Keywords:** Health sciences, Risk factors

## Abstract

Although there is no evidence that elevated rates of cesarean sections (CS) translate into reduced maternal/child perinatal morbidity or mortality, CS have been increasingly overused almost everywhere, both in high and low-income countries. The primary cesarean section (PCS) has become a major driver of the overall CS (OCS) rate, since it carries intrinsic risk of repeat CS (RCS) in future pregnancies. In our study we examined patterns of PCS, planned PCS (PPCS), vaginal birth after 1 previous CS (VBAC-1) and associated factors in Friuli Venezia Giulia (FVG), a region of North-Eastern Italy, collecting data from its 11 maternity centres (coded from A to K) during 2005–2015. By fitting three multiple logistic regression models (one for each delivery mode), we calculated the adjusted rates of PCS and PPCS among women without history of CS, whilst the calculation of the VBAC rate was restricted to women with just one previous CS (VBAC-1). Results, expressed as odds ratio (OR) with 95% confidence interval (95%CI), were controlled for the effect of hospital, calendar year as well as several factors related to the clinical and obstetric conditions of the mothers and the newborn, the obstetric history and socio-demographic background. In FVG during 2005–2015 there were 24,467 OCS (rate of 24.2%), 19,565 PCS (19.6%), 7,736 PPCS (7.7%) and 2,303 VBAC-1 (28.4%). We found high variability of delivery mode (DM) at hospital level, especially for PCS and PPCS. Breech presentation was the strongest determinant for PCS as well as PPCS. Leaving aside placenta previa/abuptio placenta/ante-partum hemorrhage, further significant factors, more importantly associated with PCS than PPCS were non-reassuring fetal status and obstructed labour, followed by (in order of statistical significance): multiple birth; eclampsia/pre-eclampsia; maternal age 40–44 years; placental weight  600-99 g; oligohydramios; pre-delivery LoS 3–5 days; maternal age 35–39 years; placenta weight 1,000–1,500 g; birthweight < 2,000 g; maternal age ≥ 45 years; pre-delivery LoS ≥ 6 days; mother’s age 30–34 years; low birthweight (2,000–2,500 g); polyhydramnions; cord prolaspe; ≥6 US scas performed during pregnancy and pre-term gestations (33–36 weeks). Significant factors for PPCS were (in order of statistical significance): breech presentation; placenta previa/abruptio placenta/ante-partum haemorrhage; multiple birth; pre-delivery LoS ≥ 3 days; placental weight ≥ 600 g; maternal age  40–44 years; ≥6 US scans performed in pregnancy; maternal age ≥ 45 and 35–39 years; oligohydramnios; eclampsia/pre-eclampsia; mother’s age 30–34 years; birthweight <2,000 g; polyhydramnios and pre-term gestation (33–36 weeks). VBAC-1 were more likely with gestation ≥ 41 weeks, placental weight <500 g and especially labour analgesia. During 2005–2015 the overall rate of PCS in FVG (19.6%) was substantially lower than the corresponding figure reported in 2010 for the entire Italy (29%) and still slightly under the most recent national PCS rate for 2017 (22.2%). The VBAC-1 rate on women with history of one previous CS in FVG was 28.4% (25.3% considering VBAC on all women with at least 1 previous CS), roughly three times the Italian national rate of 9% reported for 2017. The discrepancy between the OCS rate at country level (38.1%) and FVG’s (24.2%) is therefore mainly attributable to RCS. Although there was a marginal decrease of PCS and PPCS crudes rates over time in the whole region, accompained by a progressive enhancement of the crude VBAC rate, we found remarkable variability of DM across hospitals. To further contain the number of unnecessary PCS and promote VBAC where appropriate, standardized obstetric protocols should be introduced and enforced at hospital level. Decision-making on PCS should be carefully scrutinized, introducing a diagnostic second opinion for all PCS, particularly for term singleton pregancies with cephalic presentation and in case of obstructed labour as well as non-reassuring fetal status, grey areas potentially affected by subjective clinical assessment. This process of change could be facilitated with education of staff/patients by opinion leaders and prenatal counseling for women and partners, although clinical audits, financial penalties and rewards to efficient maternity centres could also be considered.

## Introduction

Cesarean section (CS) is one of most common major surgical procedures, life-saving both for the mother and the newborn when medically indicated^1,2^. However, as with all surgical operations, CS exposes the woman and the infant to relevant immediate as well as long term health risks, potentially affecting also the course and outcome of subsequent pregnancies^1–9^. The main obstetric complications associated with CS include maternal death, post-partum infection, uterine rupture, bladder injury, abnormal placentation, ectopic pregnancy, stillbirth, preterm birth, other^2,10–12^. Further, there is also growing evidence that CS may alter the hormonal and micro-biological physiology of the infant, compromising the flora of the gut and potentially increasing the risk of allergies by interfering with the development of the child’s immune system. These alterations seem to have a role in the enhanced risk of asthma and also on childhood obesity later in life^12^.

CS should therefore be performed only if clinically indicated, especially considering also the associated enhanced health care costs as compared to a vaginal delivery (VD)^1,13–16^. In Ireland it was estimated that a planned CS costs 739 € more than a VD, and an urgent/emergency CS (UCS) is 1,180€ more expensive than a VD^16^.

Given there is no evidence that elevated CS rates would translate into reduced maternal/child perinatal mortality, since 1985 WHO has been advocating the maintenance of the CS rate not to be higher than 10–15% in any region of the world^1,17^, although recent evidence suggests a cutoff of 19% would be more reasonable^18^. Nonetheless, CSs have been increasingly overused almost everywhere in the past decades^19,20^, becoming a pandemic phenomenon, with almost a third of women worldwide now delivering by CS^21,22^. An overall 29.7 million births (21.1% rate) occurred by CS in 2015 across the globe, almost doubling the corresponding rate of 2000 (12.1%), and an estimated 6.2 million CSs are performed in excess (without medical justification) worldwide each year^21,23^.

The primary CS (PCS) rate has become a major driver of the overall CS (OCS) rate, accounting for more than two thirds of all CSs in the USA^24–26^. Due to the uterine scar, the first CS carries intrinsic risk of repeat CS (RCS) in future pregnancies, justifying the Cragin’s dictum back in 1916 “*once a cesarean always a cesarean*”^27^. RCSs after a previous CS are significant contributors to the increase of OCS rate^27^. In 1996, the PCS rate in US was 14.5%, becoming 23.4% in 2007, thus increasing by more than 60%^28^. This trend has been continuing, as confirmed in another study at Yale-New Haven Hospital (US) during 2003–2009, reporting a 50% increase of OCS attributable to enhancing PCS rate^27^. Since it impacts the number of unnecessary CS^27^, a great deal of attention has recently been placed on the main reasons advocated by clinicians in decision-making for PCS^24^,

The planned PCS (PPCS) is an obvious target for reducing the PCS rate, to avert the vicious circle of RCSs that may subsequently arise. The critical importance of containing the planned RCS (PRCS) was endorsed by an Australian study on 81 hospitals in New South Wales, reporting that women with singleton term pregnancy, cephalic presentation and history of one previous CS constituted the strongest proportion (34%) of the OCS rate^29^. The greatest risk associated with CS is maternal death, reportedly 2.84–3.11 more likely with PPCS as compared to VD^30,31^.

Trial of labor after CS (TOLAC) is a programmed attempt to deliver vaginally for a woman with previous CS. This approach enables the opportunity to achieve a vaginal birth after CS (VBAC), a realistic option for some women with history of CS, which should be encouraged with the view of containing the number of unnecessary CS^32^.

For decades women were discouraged from VBAC, due to the risk of rupture of the previously vertically incised large uterine muscle during contractions^33^. This risk increases with number of previous CS. The introduction of the transverse lower incision diminished this risk and allowed more women to try TOLAC^32,33^. TOLAC is now widely recommended in appropriately selected and supported pregnant women with up to two transverse low-segment CS^32^. It is argued that a substantial fraction of the observed worldwide increase of planned CS in the last decades is attributable to decreasing VBAC rates^34^, which started to plummet in the mid 1990ies, in coincidence with the publication of some studies emphatizing the health risks associated with TOLAC (especially uterine rupture), with debatable evidence though^34–38^. After an initial sharp increase during 1990–1996, the VBAC rate in fact progressively declined from 32% to <10% in Massachussets (USA) during 1996–2012. The same rate importantly diminished also in the State of Hesse (Germany) from 48% to 25% during 1990–2012 and from 64% to 33% during 1990–2014 in two major maternity centres of Dublin (Ireland)^34^.

However, in addition to reduced medical expenditures, an adequate choice of TOLAC (in strict compliance with obstetric guidelines) instead of unnecessary RCS provides a number of advantages, including quicker recovery time post childbirth and a reduction of untoward maternal sequelae (hysterectomy, bowel/bladder injury, transfusion and placenta previa)^39,40^. In particular, a systematic review of 203 studies reported a significantly higher risk of maternal mortality following planned RCS (PRCS) as compared with planned TOLAC (13.4 vs. 3.8 per 100,000). Focusing on term pregnancies, a fifthfold increase of maternal mortality was observed among women receving PRCS as compared with those undergoing planned VBAC. Women undergoing PRCS also had higher risk of embolia and longer length of hospital stay following delivery (1.4 days more), as compared with those opting for TOLAC^41^.

Despite the evidence of safety and feasibility of TOLAC and the recognized health risks associated with RCS, the average rate of VBAC in the whole of Italy is still low (9–11%)^42,43^. Since Italy also has the highest CS rate (38.1%) among all European countries, and considering countries as Sweden, Finland and the Netherlands reportedly managed to reach a 45–55% VBAC rate, there is an urgent need to develop and evaluate multifaceted prenatal and perinatal interventions to effectively reduce the number of unnecessary CS in Italy, also promoting VBAC where appropriate^3,42^.

In view of the above, we examined the patterns of PCS, PPCS, VBAC and associated factors in Friuli Venezia Giulia (FVG), a region of North-Eastern Italy, during 2005–2015, to provide epidemiological figures potentially useful to design health care policies aimed at evaluating and containing the CS rate on a regional and national scale.

## Methods

### Ethics Statement

The present study is part of a project named “The Health of Mothers and Children of Friuli-Venezia Giulia (FVG) and Associated Factors”, approved by the Scientific Directorate of IRCCS “Burlo Garofolo” and submitted to the Italian Ministry of Health (MoH) on 28/10/2016 (Research Workflow ID: 2016009504). Approval to conduct the study was granted by the Regional Health Authority of FVG, a regional governmental body accountable for issuing routinely collected anonymized patients’ data to research insitutions. According to the Italian privacy law (Legislative Decree 101/2018, D.Lgs 101/2018) regional data from the Italian National Health Service (NHS) can be used for scientific purposes within the frame of approved studies/protocols. Provided sentitive information is anonymized, informed consent from patients is waived.

### The database

The present investigation employs a population-based cross-sectional design.

Data from the 11 maternity services of FVG during calendar years 2005–2015 were extracted from the Regional Repository, a database anonymously storing administrative information from the Italian NHS.

The database we analyzed included information from two sources: hospital discharge forms (ICD-9 codes) and the Certificate of Delivery Care (CEDAP, Italian acronym), a formatted questionnaire collecting clinical and personal information on women and newborns (supplementary file)^13,14,44^.

We used the following ICD-9 codes to retrieve the obstetric conditions associated with each childbirth:

- Polyhydramnios: 657.0;
- Oligohydramnios: 658.0;
- Antepartum hemorrhage, abruptio placentae and placenta previa: 641.(0-1-2-3-8-9);
- Obstructed labour (except shoulder girdle dystocia): 660.(0-1-2-3-5-6-7-8-9);
- Non-reassuring fetal status: 656.3;
- Cord prolapse: 663.0;
- Premature rupture of membranes (PROM): 658.1;
- Eclampsia/pre-eclampsia: 642.(4-5-6-7);
- Rh iso-immunization: 656.1;

The rest of data derived from CEDAP, in which delivery modes (DM) are defined as follows:

1. Vaginal delivery (VD) without forceps or vacuum extraction;
2. Planned CS or CS for failed induction;
3. CS during labour or urgent CS;
4. Forceps extraction;
5. Vacuum extraction;
6. Other forms of VD;

We considered the above category 1 as SVD, category 2 as planned CS, category 3 as UCS; categories 4, 5 and 6 were combined into instrumental vaginal deliveries (IVD). Categories 2 and 3 were incorporated to form OCS.

As a rule of thumb, when there were minor mismatches between the two sources of information we had for this study (CEDAP and HDF), priority was given to CEDAP. The original data have thus been modified as follows:

- Since shoulder presentation is incompatible with SVD, and its management by IVD is an exceptional procedure, 39 shoulder presentations delivering by SVD and 1 shoulder presentation delivering by IVD were reclassified as cephalic;

Differently from a previous study using the same database^14^, placenta previa/abruptio placenta/ante-partum hemorrage delivering by SVD (N = 133) and by IVD (N = 22) were retained in the analysis.

The 11 regional maternal facility centres were anonymized and coded by alphabetic letter from A to K. Hospitals A and B are second level maternity units, since in addition of delivering > 1,000 births per years, they are also provided with an neonatal intensive care unit, whereas the other 9 are first level. The Italian MoH recommended 1^st^ level maternity centres (defined as facilities with <1,000 annual births and/or devoid of an neonatal intensive care unit) to maintain the PCS rate under 15% and 2^nd^ level maternity units (defined as units with >1,000 yearly deliveries, equipped also with a neonatal intensive care unit) under 25%^45^. Hospital H showed the best combination of optimal DM rates, having the second lowest PCS rate (13.5%), the second lowest PPCS rate (5.4%) and the highest rate of VBAC-1 (46.5%). Moreover, despite being officially classified as 1^st^ level maternity unit, Hospital H had an overall 11,681 births during 2005–2015 (annual average of 1,062 births), consistently above the yearly threshold of 1,000 deliveries, with the exception of calendar year 2005 (N = 995 births), 2013 (N = 995 births) and 2015 (N = 879 births). Therefore, we treated hospital H as reference centre in the analysis.

### Endpoints

Using the CEDAP variable “*Number of previous CS*”, the numerator of PCS was calculated as total OCS performed in women without history of CS, whereas the numerator of PPCS was obtained by restricting PCS to planned procedures. The rates of PCS and PPCS were calculated as respective percentages out of all births.

The rate of VBAC-1 was calculated as a percentage of vaginal deliveries (VD), combining SVD as well as IVD, among all parturients with history of one previous CS. Women with history of ≥2 previous CS were excluded from the caluclation of VBAC-1, due to small numbers involved.

**Figure 1:**
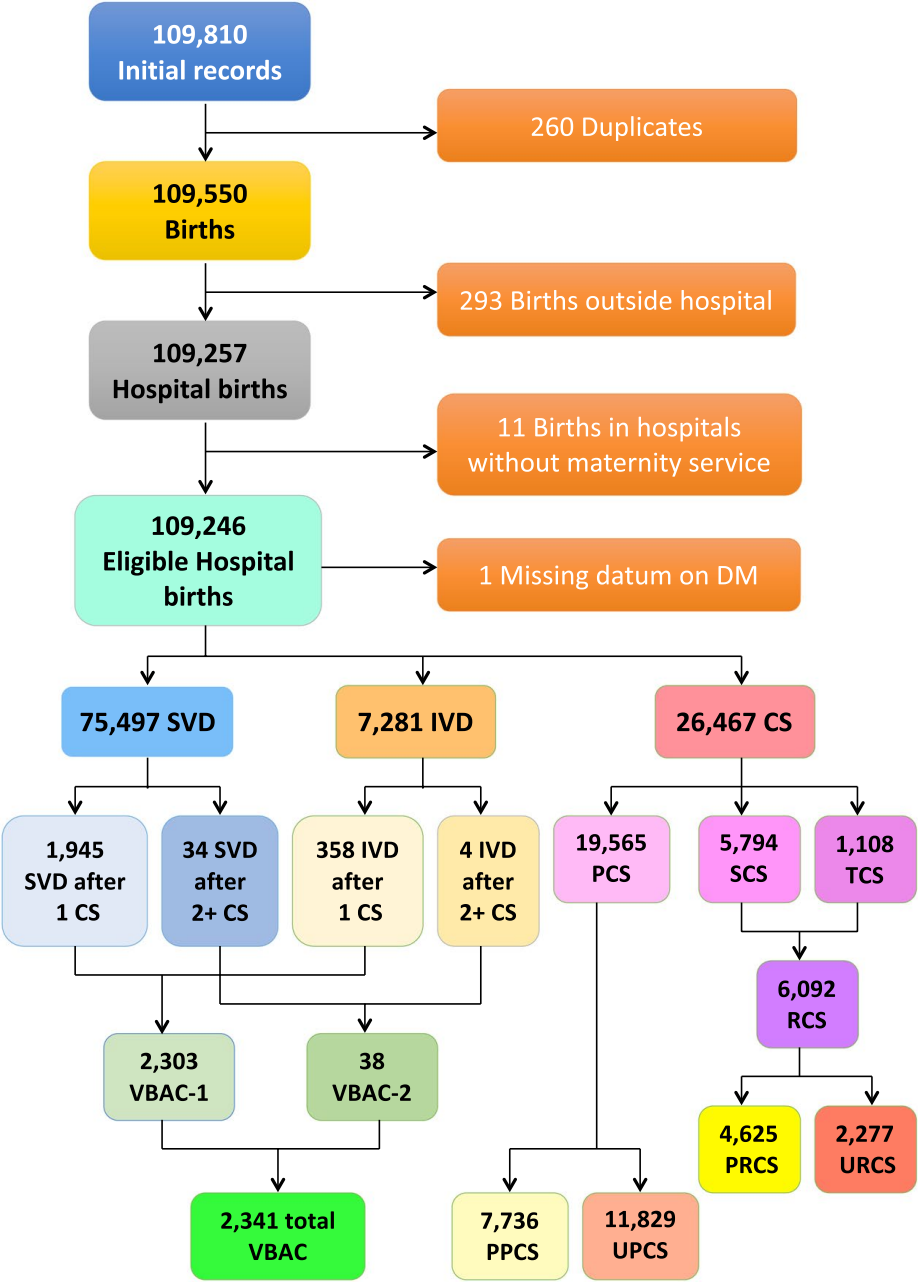
Flowchart displaying the criteria applied to the initial database to obtain the final number of records available for the analysis. SVD = Spontaneous Vaginal Deliveries; IVD = Instrumental Vaginal Deliveries; CS = Cesarean Sections; PCS = Primary CS; SCS = Secondary CS; TCS = Tertiary/more CS; RCS = Repeat CS; PPCS = Planned PCS; UPCS = Urgent/Emergency PCS; PRCS = Planned RCS; URCS = Urgent/Emergency RCS; VBAC = Vaginal Birth After CS; VBAC-1 = Vaginal Birth After 1 previous CS; VBAC-2 = Vaginal Birth After ≥ 2 previous CS; DM = Delivery Mode.

Figure 1 shows the flowchart displaying the various criteria applied to the initial database to obtain the final number of hospital records available for the analysis.

### Conceptual framework

**Figure 2:**
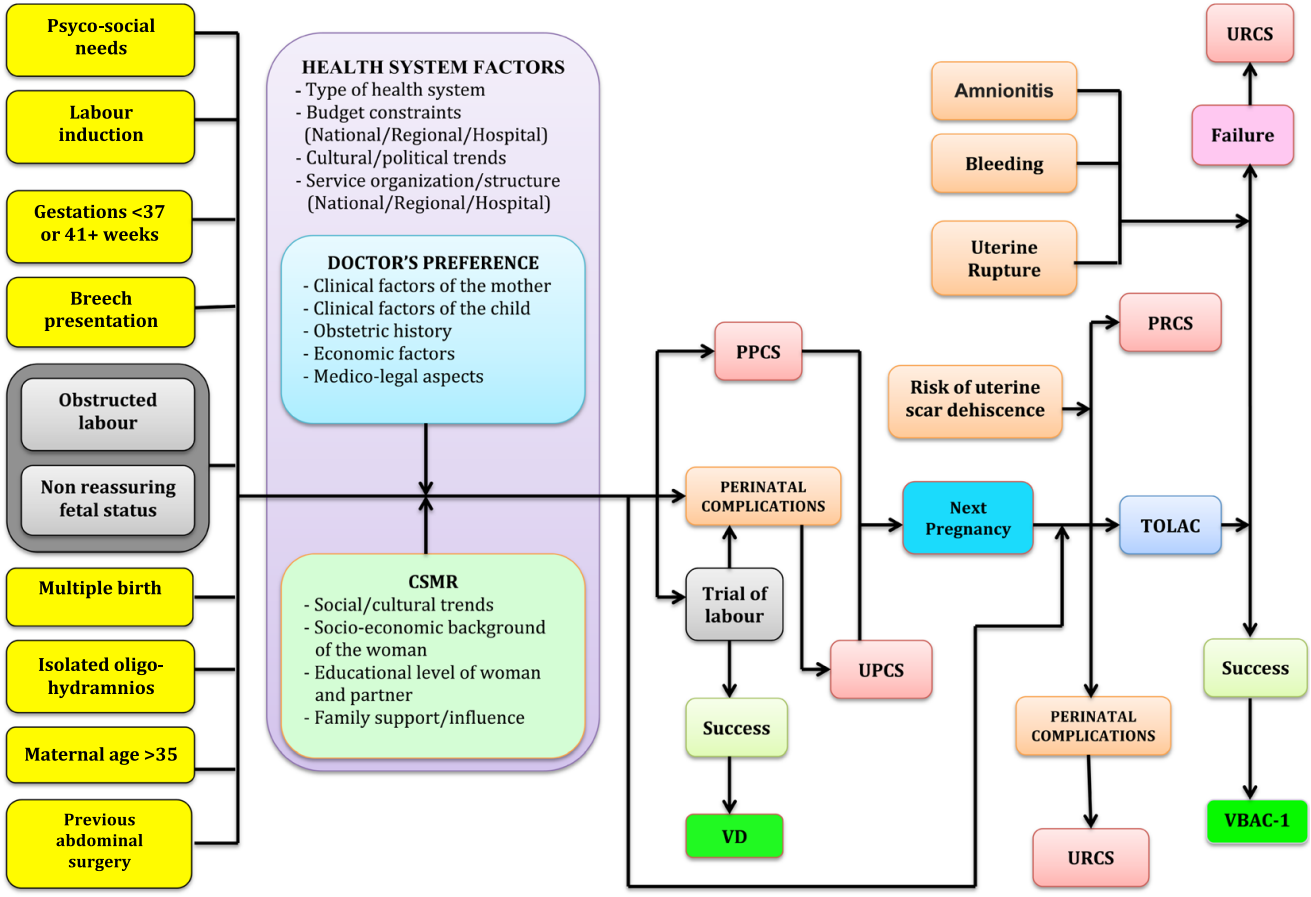
(Conceptualized by LC and GDP). Conceptual Framework explaining the relationship between various factors and delivery mode. VD = Vaginal Delivery; CS = Cesarean Sections; PPCS = Planned Primary CS; UPCS = Urgent/Emergency Primary CS; RCS = Repeat CS; URCS = Urgent RCS; PRCS = Planned RCS; TOLAC = Trial of Labour After CS; VBAC-1 = Vaginal Birth After 1 previous CS; CSMR = CS on Maternal Request.

Figure 2 displays the conceptual framework explaining the relationship between various factors considered and the three delivery modes (DM) under investigation (PCS, PPCS, VBAC-1). Five domains of potential determinants of DM were identified^13,14,44^:

1. Setting (hospitals) and timeframe (calendar year). The classes can be seen in Table 1.

**Table 1:** Total number of hospital births, rates of Overall Cesarean Sections (OCS), Primary Cesarean Sections (PCS), Planned Primary Cesarean Sections (PPCS) and Vaginal Births After 1 previous Cesarean Section (VBAC-1), by maternity centre and calendar year.

Factors	Strata	All Births (N)	OCS	PCS	PPCS	VBAC-1
			Row %			
Hospitals	A	19,059	23.2	19.9	9.0	39.4
	B	18,380	34.3	28.1	13.1	19.1
	C	8,940	20.1	15.7	8.0	31.3
	D	3,330	28.3	22.0	7.5	12.9
	E	6,673	24.4	18.7	10.0	15.2
	F	5,723	25.7	20.2	8.5	14.4
	G	9,146	15.2	12.6	4.7	45.0
	H	11,681	16.4	13.5	5.4	46.3
	I	6,047	21.6	17.7	8.1	29.7
	J	12,035	28.8	23.3	10.2	25.3
	K	8,027	21.7	16.3	8.3	29.6
Calendar year	2005	10,173	24.8	21.1	11.0	26.3
	2006	10,468	25.0	20.9	10.4	26.0
	2007	10,652	25.4	21.2	10.0	26.3
	2008	10,478	24.5	20.1	8.9	27.5
	2009	10,492	25.5	20.9	9.4	28.5
	2010	10,406	24.5	19.3	8.7	29.0
	2011	9,791	24.0	19.2	7.9	26.9
	2012	9,743	22.1	17.5	7.2	31.5
	2013	9,289	24.0	18.7	7.7	30.0
	2014	9,095	23.0	17.9	7.2	30.3
	2015	8,659	23.2	17.7	7.5	30.3
**TOTAL**		**109,246**	**24.2**	**19.6**	**8.8**	**28.4**

Number (N); row percentage (%).
2. Maternal health factors. The classes are shown in Table 2.

**Table 2:** Total number of hospital births, rates of Overall Cesarean Sections (OCS), Primary Cesarean Sections (PCS), Planned Primary Cesarean Sections (PPCS) and Vaginal Births After 1 previous Cesarean Section (VBAC-1), by maternal health factors.

Factors	Strata		All Births (N)	OCS	PCS	PPCS	VBAC-1
				Row %			
Mother’s age (years) (Missing: 32)	15–19		1,254	15.4	15.1	5.2	20.0
	20–24		9,485	17.8	15.9	5.5	32.5
	25–29		23,675	20.6	17.2	7.2	28.4
	30–34		38,381	23.5	19.1	8.6	28.9
	35–39		28,860	27.5	21.3	10.2	28.0
	40–44		7,214	35.0	28.1	14.6	27.4
	45+		345	56.2	53.1	38.3	19.4
Hypertension/diabetes (Missing: 63)	No		106,690	23.6	19.0	8.5	29.0
	Yes		2,493	51.3	45.9	22.2	12.8
Chorionic villi sampling (Missing: 6)	No		104,993	24.0	19.4	8.7	28.7
	Yes		4,247	29.4	23.0	11.3	24.2
Amniocentesis (Missing: 6)	No		91,986	23.2	18.8	8.3	29.2
	Yes		17,254	29.5	23.9	11.2	25.5
Fetoscopy (Missing: 6)	No		108,892	24.2	19.6	8.8	28.5
	Yes		348	27.3	21.8	9.0	27.3
N. obstetric checks during pregnancy (Missing: 1)	<4		20,856	26.8	22.6	11.3	26.6
	4–7		65,800	23.0	18.1	7.7	28.2
	8+		22,589	25.4	21.1	9.6	30.6
N. US scans during pregnancy (Missing: 7)	<4		19,003	17.4	13.4	4.4	36.9
	4–5		52,873	22.1	17.4	7.5	29.9
	6+		37,363	30.7	25.9	13.1	23.3
Neonatal status	Liveborn		108,944	24.2	19.6	8.8	28.4
	Stillborn		302	29.8	25.2	4.7	31.0
Pre-delivery LoS (days) (Missing: 594)	<3		103,769	22.7	18.0	7.9	29.1
	3–5		3,142	47.4	44.6	24.5	15.9
	6+		1,741	69.6	67.6	48.9	10.6
Any medical assisted fertilization (MAF)	None		108,336	23.9	19.2	8.5	28.5
	Drug induced ovulation	N = 80	910	61.5	60.8	45.1	22.9
	Intra-uterine insemination (IUI)	N = 181					
	Gamete intra-fallopian transfer (GIFT)	N = 8					
	*In Vitro* Fertilization & Embryo Transfer	N = 263					
	Intra-cytoplasmic sperm injection (ICSI)	N = 366					
	Other MAF	N = 12					

Number (N); row percentage (%).
3. Child’s clinical factors (child’s size and child’s fragility). The classes are shown in Table 3.

**Table 3:** Total number of hospital births, rates of Overall Cesarean Sections (OCS), Primary Cesarean Sections (PCS), Planned Primary Cesarean Sections (PPCS) and Vaginal Births After 1 previous Cesarean Section (VBAC-1), by clinical factors of the newborn.

Factors		Strata		All Births (N)	OCS	PCS	PPCS	VBAC-1
					Row %			
CHILD’S SIZE FACTORS	Gestational Age (weeks)	<29		563	65.5	63.6	15.6	19.2
		29–32		1,130	75.7	74.8	37.6	18.5
		33–36		6,217	51.8	47.9	25.1	19.6
		37–40		82,637	22.4	17.1	8.7	27.9
		41+		18,699	18.7	16.8	4.1	40.4
	Birthweight (gr) (Missing: 5)	<1000		525	75.1	74.0	41.3	16.4
		1,000–1,499		668				
		1,500–1,999		1,330				
		2,000–2,499		4,524	50.3	46.8	26.7	16.3
		2,500–3,999		95,954	21.7	16.9	7.8	29.6
		4,000–4,499		6,576	23.1	18.4	6.9	26.0
		4,500+		664				
	Placenta weight (gr) (Missing: 172)	<500		22,862	23.9	20.6	8.1	34.0
		500–599		35,744	19.1	15.2	6.6	34.7
		600–999		49,048	26.5	20.7	9.6	24.1
		1,000–1,500		1,420	77.9	75.1	62.6	2.7
	Child’s size *	SGA		9,122	32.1	28.1	13.0	28.5
		AGA		88,138	23.2	18.5	8.4	29.1
		LGA		11,986	25.5	20.7	8.8	23.6
CHILD’S FRAGILITY FACTORS	Apgar score 1 minute	<7		6,807	43.9	41.1	13.4	26.9
		7+		102,439	22.9	18.1	8.5	28.5
	Apgar score 5 minute	<8		2,386	48.6	45.7	12.7	25.0
		8+		106,860	23.7	19.0	8.7	28.5
	Multiple births (Missing: 898)	Singleton	Female	51,806	22.7	17.9	7.7	28.9
			Male	54,797				
		Twins or more		1,745	87.3	86.7	76.9	6.5

Number (N); row percentage (%).

^*^SGA = Small for Gestational Age; AGA = Appropriate for Gestational Age; LGA = Large for Gestational Age.
4. Socio-demographic background and obstetric history. The corresponding classes are displayed in Table 4.

**Table 4:** Total number of hospital births, rates of Overall Cesarean Sections (OCS), Primary Cesarean Sections (PCS), Planned Primary Cesarean Sections (PPCS) and Vaginal Births After 1 previous Cesarean Section (VBAC-1), by socio-demographic and obstetric history factors.

Factors		Strata			All Births (N)	OCS	PCS	PPCS	VBAC-1
						Row (%)			
SOCIO-DEMOGRAPHIC FACTORS	Father’sage (years) (Missing: 1,949)	15–19			199	10.1	10.2	3,3	100.0
		20–24			2,798	17.2	15.8	4.9	27.9
		25–29			12,982	20.8	18.2	7.1	28.7
		30–34			31,601	22.7	18.8	8.1	28.2
		35–39			34,560	24.5	19.2	8.9	28.5
		40–44			17,866	27.4	21.0	10.0	28.7
		45–49			5,353	30.5	24.3	11.9	28.6
		50–54			1,361	30.9	25.8	13.7	27.6
		55+			577	31.2	26.8	15.9	27.3
	Mother’s nationality (Missing:116)	EU		Italian	86,083	24.0	19.6	9.0	28.9
				Non-Italian	5,983	20.8	16.6	6.8	25.6
		Non-EU			17,064	26.5	20.2	8.3	27.1
	Marital status (Missing: 8,155)	Not married			12,036	23.9	21.8	9.5	27.3
		Married			70,340	24.4	19.1	8.7	29.1
		Separated			1,136	32.1	26.0	13.8	29.2
		Widow			82				
		Divorced			669				
		Living together			16,846	22.5	19.6	8.0	31.0
	Mother’s education (Missing: 24)	University/more			29,150	23.8	19.9	9.2	30.5
		Secondary			52,988	23.8	19.4	8.5	28.4
		Junior secondary			25,107	25.3	19.4	8.8	26.7
		Primary/none			1,977	28.3	21.0	8.8	27.5
	Father’s education (Missing: 6,772)	University/more			18,542	24.4	20.1	9.5	29.6
		Secondary			51,356	23.7	19.3	8.5	29.8
		Junior secondary			30,767	24.4	19.1	8.5	27.9
		Primary/none			1,809	26.3	19.8	8.5	27.9
	Mother’s occupation (Missing: 448)	Unemployed/student/housewife			34,144	24.8	18.8	8.0	27.8
		Self-employed/Enterpreur			9,037	25.0	20.7	9.5	27.6
		Manager			2,145	27.0	22.6	10.8	25.3
		Employed (Clerk)			31,002	23.3	19.5	9.2	29.8
		Blue Collar			12,836	25.0	20.4	9.3	27.7
		Employed (other)			19,634	23.6	19.5	8.6	29.3
	Father’s occupation (Missing: 7,145)	Unemployed/student/housewife			3,722	27.2	21.4	8.8	23.9
		Self-employed/Enterpreneur			22,100	23.4	18.9	8.6	31.7
		Manager			3,338	28.9	23.4	12.1	21.0
		Employed (Clerk)			22,537	23.3	19.3	9.0	31.1
		Blue Collar			32,812	24.4	19.1	8.3	28.2
		Employed (other)			17,592	23.7	19.3	8.5	28.5
	Consanguinity	No			109,099	24.2	19.6	8.8	28.4
		Yes			147	19.1	17.0	10.0	33.3
OBSTETRIC HISTORY FACTORS	N. previous livebirths		0		58,217	25.0	24.9	10.0	16.7
			1		39,805	23.3	12.2	6.2	24.4
			2		8,644	24.7	12.0	5.7	51.6
			3		1,820	22.6	11.7	5.8	52.3
			4+		755	17.4	9.6	4.7	61.5
	N. previous stillbirths		0		108,502	24.1	19.5	8.7	28.4
			1+		744	44.4	29.6	17.2	30.1
	N. previous Pre-term babies (Missing: 1,144)		0		105,774	24.0	19.7	8.8	27.9
			1		2,041	35.1	14.1	7.0	33.6
			2+		287	44.3	21.0	9.3	43.5
	N. previous intentional abortions		0		100,653	24.2	19.5	8.8	28.2
			1		7,038	246	20.0	8.8	31.6
			2+		1,555	28.2	22.1	9.2	31.3
	N. previous spontaneous abortions		0		92,694	24.0	19.6	8.7	28.2
			1		12,555	24.5	18.5	8.6	30.2
			2		2,897	27.8	21.0	9.9	29.5
			3+		1,099	34.7	26.2	13.5	24.7
	N. previous neonatal deaths		0		108,923	24.2	19.6	8.8	28.4
			1+		323	42.4	16.7	10.2	33.7

Number (N); row percentage (%).
5. Obstetric conditions, shown in Table 5: oligohydriamnios, polyhydramnios, eclampsia/pre-eclampsia, placenta previa/abruptio placenta/ante-partum hemorrhage, non-reassuring fetal status, congenital malformations at birth, cord prolapse, PROM, RH iso-immunization, obstructed labour (except shoulder girdle dystocia), labour analgesia, labour induction, fetal presentation.

**Table 5:** Total number of hospital births, rates of Overall Cesarean Sections (OCS), Primary Cesarean Sections (PCS), Planned Primary Cesarean Sections (PPCS) and Vaginal Births After 1 previous Cesarean Section (VBAC-1), by obstetric factors.

Factors	Strata	All Births (N)	OCS	PCS	PPCS	VBAC-1
			Row %			
Oligohydramnios (Missing: 751)	No	105,891	23.8	19.1	8.6	28.8
	Yes	2,604	39.3	36.3	16.6	11.0
Polyhydramnios (Missing: 751)	No	108,060	24.1	19.5	8.7	28.6
	Yes	435	52.9	46.3	22.4	13.8
Eclampsia/pre-eclampsia (Missing: 751)	No	107,127	23.6	18.9	8.6	28.8
	Yes	1,368	69.4	67.4	34.6	8.7
Placenta previa/abruptio placenta/ante-partum hemorrage (Missing: 751)	No	107,213	23.5	18.8	8.5	28.8
	Yes	1,282	87.7	86.9	65.3	6.8
Non reassuring fetal status (Missing: 751)	No	105,798	23.2	18.4	8.7	28.5
	Yes	2,697	64.9	64.5	15.3	286
Fetal anomalies (Missing: 751)	No	108,474	24.2	19.6	8.8	28.5
	Yes	21	38.1	27.8	13.3	0
Cord prolapse (missing: 751)	No	108422	24.2	19.5	8.8	28.5
	Yes	73	91.8	91.0	0	0
Premature rupture of membranes (Missing: 751)	No	95,699	24.3	19.4	9.4	28.3
	Yes	12,796	23.6	20.6	3.9	30.8
Rh Iso-immunization (Missing: 751)	No	108,399	24.2	19.5	8.8	28.5
	Yes	96	43.8	39.1	24.3	12.5
Obtructed labour (but shoulder dystocia) (Missing: 751)	No	105,295	23.0	18.2	8.6	28.5
	Yes	3,200	63.1	62.5	16.9	27.5
Labour analgesia (Missing: 184)	No	89,536	25.8	20.4	10.3	25.9
	Yes	19,526	16.5	15.9	2.0	65.1
Labour mode (Missing: 276)	Spontaneous	69,483	8.6	7.2	0.5	64.3
	Stimulated	6,786	14.6	14.2	1.3	72.6
	Induced	17,010	22.3	21.8	6.3	47.0
	No labour	15,691	100	100	100	0
Presentation (Missing: 181)	Cefalic	103,611	20.5	15.7	5.9	29.8
	Breech	5,288	92.5	92.6	88.2	7.1
	Shoulder	126	100	100	100	0

Number (N); row percentage (%).

### Statistical analysis

We employed a logistic regression approach using as outcome the variable 1/0 (1 = each DM vs. 0 = rest) to investigate the impact of various determinants on each three examined DM (PCS, PPCS, VBAC-1). Significant terms to be retained in the final model were selected by backward stepwise procedure.

Labour mode was excluded from all final multiple regression logistic models, because its stratum “*no labour*” comprised only PCS, thus generating collinearity issues with various DM outcomes.

Likewise, shoulder presentations (fetal transverse lies) were also excluded from all final multivariable models, since they were all delivered by CS.

Apgar score at 1 and 5 minutes were excluded since they were postnatal clinical parameters.

The following significant factors were dropped from the final multivariable logistic regression model despite being significant at bivariate analysis adjusted only for hospital, since they were affected by a substantial number of missing values:

- PCS: marital status (p < 0.001); father’s education (p = 0.003);
- PPCS: marital status (p < 0.001); father’s education (p = 0.003); father’s occupation (p = 0.003);
- VBAC-1: father’s occupation (p < 0.001).

As the percentage of missing values was less than 10% for all factors included in all 3 multivariable logistic models, complete case analysis was adopted.

Results were obtained by comparing each stratum specific estimate with the reference category and were expressed as odds ratio (OR) with 95% confidence interval (95%CI). Considering the large number of statistical tests performed in the multivariable logistic regression models, some p-values could have been significant by chance. Therefore, we employed as further selection criterion the procedure proposed by Benjamini-Hochberg (BH), setting the false discovery rate (FDR) at 5%^46^.

Stata 14.3 (College Station, Texas, USA) was employed for the analysis.

## Results

### Descriptive results

As can be seen from Fig. 1, in FVG during 2005–2015 there were 75,497 SVD (rate of 69.1%), 7,281 IVD (rate of 6.7%) and 26,467 CS (rate of 24.2%).

PCS were 19,565 (19.6% of all women without history of CS). Secondary CS (SCS) were 5,794 and tertiary/more CS (TCS) were 1,108, for a total 6,092 RCS (24.9% out of OCS). Total PPCS were 7,736 (8.8% among women without history of CS), urgent/emergency PCS (UPCS) were 11,829, planned RCS (PRCS) were 4,625 and urgent/emergency RCS (URCS) were 2,227. As can be seen, whilst the proportion of UCS was predominat among PCS (UPCS/PCS = 60.5%), for RCS the latter figure completely reversed (PPCS/RCS = 75.4%).

We observed 1,945 SVD and 358 IVD following one previous CS, for a total 2,303 VBAC-1, a rate of 28.4% (=2,303/8,097) out of women with history of one single previous CS. The VBAC rate on women with history of ≥2 previous CS was 3.3% (=38/1,146), considering all women with at least 2 previous CS as denominator.

Table 1 shows the rates of PCS, PPCS and VBAC-1 by calendar year and maternity centre. In this table (and in the following descriptive Tables 2–5), the overall number of births is displayed to highlight the relative weight of each factor/stratum. The crude rates of OCS, PCS, PPCS and especially VBAC-1 showed marked variability by maternity centre. Among 2^nd^ level maternity units (A, B), during 2005–2015 centre B (28.1%) was the only one surpassing the 25% PCS benchmark recommended by the Italian MoH. Among 1^st^ level maternity units, all but G and H overtook the 15% PCS rate cutoff, although generally by small margin. As explained above, hospital H (the reference) had a 13.5% PCS rate. There was a progressive decrease in the PCS and PPCS crude rates from 2005 to 2015; by contrast, the regional crude rates of VBAC-1 showed a progressively increasing trend during the study period.

Table 2 shows the crude rates of PCS, PPCS and VBAC-1 by maternal health factors. The PCS rate increased with maternal age, pre-delivery LoS, hypertension/diabetes, higher number of ultrasound (US) scans performed during pregnancy, stillbirth, amniocentesis and chorionic villous sampling. Similar mitigated patterns were observed also for the PPCS rate, although reversed figures (lower rates) were found for stillbirth. By contrast, the VBAC-1 rate was slightly higher for maternal age 20–39 years, increased with number of obstetric checks during pregnancy, decreased considerably with increasing pre-delivery LoS and with number of US scans performed during pregnancy, and was higher for hypertension/diabetes, amniocentesis and chorionic villous sampling.

Table 3 shows the crude rates of PCS, PPCS and VBAC-1 by clinical factors of the newborn. The PCS rate decreased dramatically with higher gestational age, increased significantly with birthweight and placenta weighing 1,000–1,500 g and was considerably higher for multiple birth, Apgar score at 5 minute < 8 and Apgar score at 1 minute < 7. By contrast, apart from similar figures for multiple birth, the rate of PPCS was higher for gestational age 29–36 weeks, diminished notably with birthweight and did not vary with Apgar score at 1 as well 5 minutes. The VBAC-1 rate increased with gestational age, birthweight and singleton pregnancies, whereas it diminished with higher placenta weight.

Table 4 displays the crude rates of PCS, PPCS and VBAC-1 by socio-demographic and obstetric history factors. All in all, the rates of the various DM were relatively stable across the classes of variables displayed in Table 4. The PCS rate increased with father’s age and was higher among separated/widow/divorced women. An isolated low rate of VBAC was found among fathers that were managers. Among obstetric history factors, the rate of PCS and PPCS diminished with higher number of previous livebirths, but was higher with history of stillbirth and neonatal deaths and increased with number of previous spontaneous abortions. Conversely, the rate of VBAC-1 increased with higher number of previous livebirths, no history of stillbirth and higher number of pre-term babies.

Table 5 displays the distribution of the crude rates of the three DM by obstetric factors. The PCS rate was remarkably and consistently higher across all obstetric conditions, apart from PROM. Likewise, with the exception of non-reassuring fetal status and obstructed labour, the PPCS rate was more frequent in all obstetric conditions listed in Table 5. Reverse figures were found for VBAC-1, with highest crude rates observed for no labour induction. Parturients administered labour analgesia instead showed the second highest VBAC-1 rate.

### Outcome results

Supplementary Table 1 displays the results of the multiple logistic regression models, one for each DM (PCS, PPCS and VBAC-1).

Stronger associations for PCS were found for (in decreasing order of BH p value): breech presentation; placenta previa/abruptio placenta/ante-partum haemorrhage; non-reassuring fetal status; obstructed labour; multiple birth; eclampsia/pre-eclapmpsia; mother’s age 40–44 years; placental weight 600–999 g; oligohydramnios; pre-delivery LoS 3–5 days; mother’s age 35–39 years; placenta weight 1,000–1,500 g; very low birthweight (VLBW, birthweight <2,000 g); maternal age ≥ 45 years; pre-delivery LoS ≥ 6 days; mother’s age 30–34 years; low birthweight (2,000–2,500 g); polyhydramnios; cord prolapse; ≥6 US scans during pregnancy; pre-term gestation (33–36 weeks); maternal age 25–29 years; lower maternal education; 4–5 US scans in pregnancy; non-EU nationality of the woman; gestation ≥ 41 weeks; birthweight ≥4,000 g; hypertension/diabetes and Rh iso-immunization.

PPCS were more likely with (in descending order of BH significance): breech presentation; placenta previa/abruptio placenta/ante-partum haemorrhage; multiple birth; pre-delivery LoS ≥ 3 days; placental weight 1,000–1,500 g; mother’s age 40–44 years; placental weight >600 g; mother’s age ≥ 45 years; ≥6 US scans performed in pregnancy; mother’s age 35–39 years; oligohydramnios; eclampsia/pre-eclampsia; mother’s age 30–34 years; birthweight <200 g; polyhydramios and pre-term gestation (33–36 weeks).

Factors associated with VBAC-1 were administration of labour analgesia (strong association) and less importantly placental weight < 500 g as well as gestations > 41 weeks. Conversely, VBAC-1 were less likely with large placentas (≥600 g), breech presentation, ≥6 US scans performed during pregnancy; placenta previa/abruptio placenta/ante-partum haemorrhage; oligohydramnios pre-delivery LoS ≥ 3 days and low birthweight (2,000–2,500 g). 

Supplementary Table 2 displays the hospital variability on adjusted rates of PCS, PPCS and VBAC-1. The listed risk estimates of Supplementary Table 1 and 2 are controlled for the same factors indicated in the legend at the bottom of both tables, since they were obtained from the same multiple logistic regression models. All hospitals were more likely to deliver PCS than the referent (centre H), and all maternity units but G were more likely to perform PPCS and less likely to deliver by VBAC-1 than the reference.

In maternity units B, D, E, I and J the adjusted rates of PCS ad PPCS were both importantly higher and accompained by substantially lower corresponding adjusted rates of VBAC-1, although in centres B and D the risk of PCS was more significant than PPCS. Conversely, the level of significance of the respective risk estimates were higher for PPCS than PCS in centres A, C, F, J and particularly E and K.

## Discussion

### Key findings

In the whole FVG during 2005–2015:

- OCS were 24,467 (a rate of 24.2%), well below the corresponding national figure most recently reported from the entirety of Italy (38.1%)^3^.
- PCS were 19,565 (an overall regional rate of 19.6% during the entire study period, progressively declining from 21.1% in 2005, to 19.3% in 2010 and 17.7% in 2015), hence well below the corresponding national picture reported for 2010 (29.0%) and slightly under the most recent figure of 22.2% reported for 2017 for the whole Italy^43^. The main factors associated with PCS were (in descending order of BH significance): breech presentation; placenta previa/abruptio placenta/ante-partum haemorrhage; non-reassuring fetal status; obstructed labour; multiple birth; eclampsia/pre-eclampsia; mother’s age 40–44  years; placental weight 600–999 g; oligohydramnios; pre-delivery LoS 3–5 days; maternal age 35–39 years; placenta weight 1,000–1,500 g; very low birthweight (VLBW, birthweight <2,000 g); maternal age ≥ 45 years; polyhydramnios; cord prolapse; ≥6 US scas during pregnancy; pre-term gestation (33–36 weeks); maternal age 25–29 years; lower maternal education; gestation <29 weeks; non-EU nationality of the woman; gestations ≥41+ weeks; birthweight ≥ 4,000 g; hypertension/diabetes and Rh iso-immunization.

Among 2nd level maternity units (A, B), centres B (28.1%) surpassed the PCS benchmark of 25% recommended by the Italian MoH during the study period. Among 1st level maternity units (C, D, E, F, G, H, I, J, K), all but G overtook the respective 15% PCS rate cut-off, although generally by small margin.

- PPCS were 7,736 (a rate of 8.8%). Principal determinants were (in descending order of BH significance): breech presentation; placenta previa/abruptio placenta/ante-partum hemorrage; multiple birth; pre-delivery LoS ≥ 3 days; placental weight ≥ 600 g; mother’s age ≥ 45 years; ≥ 6 US scans performed in pregnancy; mother’s age 35–39 years; oligohydramnios; eclampsia/pre-eclampsia; mother’s age 30–34 years; birthweight < 2,500 g; polyhydramnions and pre-term gestation (33–36 weeks).
- VBAC-1 on women with history of one previous CS were 2,303 (a rate of 28.4%). By including also VBAC-2 on women with 2+ previous CS (N = 38) the overall VBAC rate in the region would slightly reduce to 25.3%, almost three times the corresponding national rate (9%) reported in 2017 for the entire Italian country^43^. VABC-1 were more likely in parturients receiving labour analgesia, with placental weighing < 500 g and gestations  ≥41 weeks.

Since the overall rate of PCS in FVG during 2005–2015 was 19.6% (21.1% in 2005, 19.3% in 2010, 17.9% in 2014 and 17.7% 2015), and the respective national estimates were 29.0% in 2010 and 22.2% in 2017, the difference between the OCS rate of FVG during the whole study period (24.2%) as compared to corresponding recent reports for the entire Italy (38.1%) can only be attributable to RCS. The critical importance of PCS on the risk of RCS was confirmed in the present study, considernig that whilst UCS were more prevalent than planned CS among PCS (UPCS/PCS = 60.5%), the latter figure fully overtuned amongst RCS (PPCS/RCS = 75.4%).

Although the crude rates of PCS and PPCS were progressively diminishing over the years, and the crude VBAC-1 rate was increasing, only the adjusted rate of PPCS significantly decreased over time in the entire region. We instead observed high variability of crude and adjusted rates of all three examined DM at the hospital level, especially for PCS and PPCS, with all centres more likely to perform PCS than the reference (centre H) and all hospitals but G more likely to deliver PPCS a well as less likely to deliver VBAC-1.

### Strengths and limitations

Strengths of this study have been reported elsewhere^13,14,44^. There are, however also some limitations.

First, we did not have information on maternal request, an important indication for PPCS^47^. Although the International Federation of Gynecology and Obstetrics considers it unethical to perform a planned CS without medical indications, growing consensus has emerged among obstetricians on the importance of informing and counselling the woman regarding risks and benefits of a planned CS, involving also her family^47,48^. In addition to immediate complications such as infections, hemorrhage and visceral injury, PPCS exposes the woman to other health risks in the long-run, especially in subsequent pregnancies: uterine rupture, placenta previa/placenta accreta/abruptio placenta and ectopic pregnancy^47,49,50^. Recent obstetric guidelines from the National Institute for Health and Care Excellence (NICE) of the United Kingdom (UK) clarify this contentious issue. A maternal request for CS signifies that the woman has requested a CS in absence of any identifiable medical or obstetric indication. Such requests should be reviewed by the consultant obstetrician, and if deemed beneficial, other members of the obstetric team (midwifes, anaesthesiologist) should provide their input^51^. This counselling aims to define (and document) the underlying reasons for the maternal request, establishing whether the woman is fully aware of the pros and cons (especially in terms of health implications) of a CS as compared to a VD. Women requesting a CS for fear or extreme anxiety about VD should be referred to a specialist perinatal mental health practitioner to address their psychological status and provide adequate support. Tocophobia is considered a mental health disorder for which CS is medically indicated to avoid the psychological harm related with a VD. Women still reluctant to opt for a VD despite full discussion on risks/benefits of CS and the offer of support against anxiety associated with childbirth should be granted a planned CS, in compliance with their request^51^.

A second limitation of the present study is the lack of documentation on number of TOLAC offered and VBAC successfully achieved. The potential benefits and disadvantages of both TOLAC and PRCS should be discussed, and these discussions should be documented on medical records. All in all, whilst a CS may provide some immediate benefits mainly to the woman and the obstetrician, a VBAC may be more in the interest of the child, especially for future pregnancies: although VBAC carries some risk in the short term, it is certainly rewarding in the long run. These discussions should consider the individual characteristics of the parturient affecting the likelihood of complications associated with a TOLAC and a PRCS. VBAC checklists and calculators are available to provide more specific counselling on the chance of a successful TOLAC^32^. Therefore, we suggest updating the CEDAP questionnaire to include also information on whether the woman is affected by tocophobia, whether a CS was performed on maternal request and whether a TOLAC was offered, attempted and the relative outcome (successful VBAC or UCS). Further relevant information to be collected in future by CEDAP would be hystory of abdominal surgery and whether an external version in case of breech presentation was offered, attempted and the relative outcome (VD, PCS or even UCS).

The calculation of the PPCS rate may have been slightly inflated, since the respective numerator obtained from CEDAP comprised both planned CS and CS performed after failed induction, distinguished categories which were impossible to disentangle though. However, the crude rate of labour induction was 15.6% out of all births in FVG during 2005–2015^14^, hence the impact of CS for induction failure on the calculation of the PPCS rate was likely marginal in our study.

We consider the VBAC rates on women with history of only one previous CS in the analysis, since the number of VBAC on women with 2+ previous CS was negligible (N = 38). However, in case of favourable obstetric conditions, TOLAC is still possible also in women with 2 previous CS and with an interval of less than 6 months between the last CS and the conception of the subsequent pregnancy^32,52^.

Some available variables (birthweight, placenta weight) reflected actual postnatal measurements rather than fetal estimations, thus potentially hampering to some extent the interpretation of the relative findings in terms of prenatal decision making on DM.

Further relevant risk factors for CS as body mass index (BMI), smoking status, physical exercise and Bishop index were not available from CEDAP for years 2005–2015^53^.

### PCS and PPCS

The PPCS rate in FVG during 2005–2015 was probably slightly lower than that found in the present investigation (8.8%) since, as explained in the methods, in CEDAP planned CS and CS due to failed induction were assembled in the same DM category.

Albeit the effect of calendar year was significant only for PPCS at multivariable logistic analysis, the crude rates of PCS and PPCS were both progressively diminishing over the years in the entire region, hinting at possible growing awareness (among society and the medical community) that a proportion of these surgical obstetric procedures are unnecessary and could be averted.

The most relevant factors, associated with balanced significance both with PCS and PPCS in the present investigation, were breech presentation and placenta previa/abruptio placenta/ante-partum haemorrhage. Malpresentation was reported as third indication for performing a CS in a recent study on 228,562 parturients affiliated with the Consortium on Safe Labor in the USA from 2002 to 2008^54^ Boyle *et al*. External version in near term pregnancies with breech presentation for selected parturients and vaginal breech delivery are highly encouraged by the American College of Obstetricians and Gynecologists (ACOG) as strategies to contain the number of redundant PCS^25,55,56^.

Following breech presentation and placenta previa/abruptio placenta/ante-partum haemorrhage, the most important factors more strongly associated with PCS than PPCS in the present study were non-reassuring fetal status and obstructed labour. PPCS were a sub-category of PCS not including UCS. It may be argued that the corresponding PCSs for the latter two conditions were predominantly urgent obstetric procedures. Obstructed labour and non reassuring fetal status are rated grey areas potentially affected by subjective diagnosis and misclassification^28,57,58^. These subjective assessments are reportedly influencing a large fraction of PCS^28^. Suspected fetal asphyxia is diagnosed by electronic fetal heart rate monitoring (EFM) during labour, its interpretation is rather subjective and varies extensively by provider^54^. Since EFM has low intrinsic specificity, most fetuses diagnosed with asphyxia by EFM are instead frequently in good condition and fit to bear the stress of labour^59^.

Obstructed labour (highly linked with labour induction) is of particular concern, since 38.9% (=6,823/19,565) PCS in our investigation were performed in primigravidas at term with a singleton fetus in cephalic presentation. As with non-reassuring fetal status, the diagnosis of obstructed labour also varies by practice pattern, especially in terms of number of cervical checks, assessments of uterine contractions and evaluations of labour timing^28^.

Zhang obtained a completely different labour curve than Friedman’s on 62,415 parturients with singleton pregnancy, cephalic presentation, physiological outcome and VD^60^. The latter study concluded that latent stage labour requires time, and delayed admission to its active phase may curb the cascade ultimately leading to a CS. This time serves to attain a satisfactory cervical dilation and should be distinguished from the active induction on an already ripened cervix^60^. In another study on 38,484 PCS among 228,562 deliveries affiliated with Consortium of Safe Labour from 2002 to 2008 in the USA, 42.6% primiparas and 33.5% multiparas underwent PCS for failure of progression with cervical dilation < 6 cm^54^. Provided there is reassuring fetal as well as maternal status, CS should be carefully avoided until completion of this latent stage, ideally until a 6 cm cervix diameter is accomplished^54,60^.

According to the ACOG, adequate timing should also be allowed for second stage labour: at least 2 hours pushing in multiparas and at least 3 hours in nulliparas^25^. In a recent randomized controlled trial (RCT) an extra hour pushing given to women achieved a significant reduction in the number of CS as compared to ordinary labour management^61^. Among women diagnosed with prolonged (second stage) obstructed labour, 20.5% primiparas were delivered < 3 hours and multiparas < 2 hours after complete cervical ripening, with only 1.1% women given a trial for instrumental vaginal delivery (IVD). Therefore, allowing adequate time during second stage labour and performing IVD where appropriate may have a major effect on decreasing the number of unnecessary PCS in case of obstructed labour^54^.

Subsequent significant factors, still more strongly associated with PCS than PPCS in this study were eclampsia/pre-eclampsia, oligohydramnios, birthweight < 2,000 g and pre-term gestations (33–36 weeks). Whilst eclampsia/pre-eclampsia and very low birthweight (VLBW) are critical conditions often requiring emergency obstetric care^14^, isolated oligohydramnios at term was found to be a risk factor for labour induction, CS and short-term neonatal morbidity in a recent systematic review on 35,999 women, with 2,414 (6.7%) of them being affected by oligohydramnios^62^. However, the health risks associated with isolated oligohydramnios diminish with increasing gestational age, being relatively lower in the last trimester^62^. In the present investigation, the clear majority of PCS (53.3% = 478/893) among women with oligohydramnios were performed for term pregnancies (37–40 weeks), and 91.2% (=815/893) in gestations ≥ 33 weeks. As to pre-term gestations, the appropriate DM should be discusssed between the woman and the obstetrician^14^.

The following conditions associated with high yet more balanced significance between PCS and PPCS were multiple birth, maternal age ≥ 35, placental weight ≥600 g, pre-delivery LoS ≥ 3 days and ≥ 6 US scans performed during pregnancy.

In case of monochorial twins, large placentas may mask the effect of multiple birth. Obstetric guidelines recommend VD for vertex presenting twins. In our study 76.9% (=714/929) multiple births were delivered by PPCS. However, 25% PCS on women carrying twins are reportedly performed with both twins in cephalic presentation and 25% with cephalic presentation of the leading twin. PCS represents for some patients and clinicians an option to avert CS for the non-cephalic second twin after VD of the first. Training obstetricians on vaginal extraction by external version for breech presentation is recommended to decrease the number of unnecessary PCS with a non-cephalic second twin^63^. However, big placentas may also reflect macrosomia, a frequent contributor to obstructed labour and a risk factor for failed TOLAC^32^. Although suspected macrosomia is an increasing indication for PCS, congenital malformations at birth were not associated with PCS or PPCS in our study and the level of significance of birthweight ≥ 4,000 g and hypertension/diabetes on the PCS risk was relatively lower^28^.

As to maternal age, women older than 35 are more likely to be affected by obesity, hypertension and diabetes, which in turn have an impact on fetal anomalies^64^. Moreover, advanced maternal age increases the risk of spontaneous abortion, pre-term delivery and perinatal bleeding^65,66^. However, decision to perform a PPCS should consider the actual health status of the woman, rather than just age^64^.

Although pre-delivery LoS ≥ 2 days and ≥ 3 US scans performed during pregnancy were significant factors associated with both PCS and PPCS in the present study, the respective associations were stronger for PPCS. Increasing pre-delivery LoS and higher number of US scans during pregnancy are likely signs of high risk gestation courses driving to programmed CS. By contrast, polyhydramnios and cord prolapse were factors more importantly associated with PCS. Cord prolapse, a critical condition often requiring UCS, can be determined by malpresentations, fetal manipulations and PROM, all conditions which in turn may be influenced by polyhydramnios^67^. Nonetheless, when not associated with obstetric circumstances that may require CS, such as pre-term labour, PROM, malpresentation, hypertensive disorder or macrosomia^68,69^, fetuses with isolated polyhydramnios can be delivered vaginally^68,69^.

There were other significant factors mainly associated with PPCS (as for instance pre-term gestation, birthweight < 2,500 g and paternal age ≥ 55 years), but their level of significance was lower and the interpretation of the relative findings could have been hampered by lack of information on maternal request, an important driver of PPCS. It is worth noting that the number of CS performed on maternal request or on doctor’s preference without medical indication is growing also because CS is perceived safer than VD in a range of conditions, including history of abdominal surgery and bowel resection for endometriosis^70–73^.

### TOLAC and VBAC

**Figure 3:**
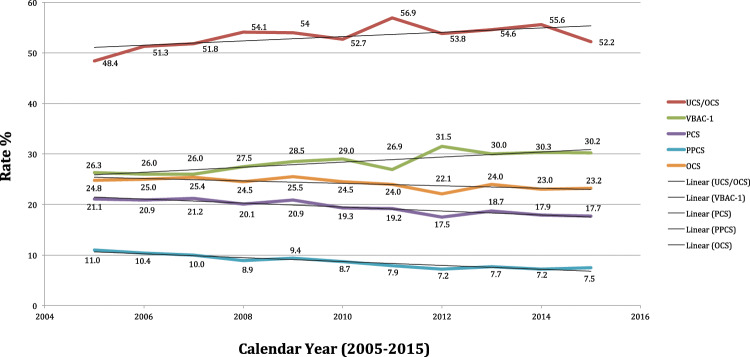
Crude rates of Overall Cesarean Sections (OCS), Primary Cesarean Section (PCS), Planned Primary Cesarean Sections (PPCS), Vaginal Birth After 1 Cesarean (VBAC-1) and Urgent/Emergency Cesarean Sections (UCS) out of OCS (UCS/OCS) in Friuli Venezia Giulia, during 2005–2015.

The VBAC-1 rate found in FVG during 2005–2015 was 28.4% on women with history of one single previous CS. Considering also VBAC on women with ≥2 previous CS, the respective rate slightly reduced to 25.3% (by including all women withat least 1 previous CS as denominator), about three times the most recent national estimates reported for the entire Italy (9–11.4%)^42,43^. Since the average cost associated with a CS is 960€, an increase of the VBAC rate at national level from 10% to 30% (hence near figures achieved in FVG during 2005–2015) is estimated to translate into 13.8 million € annual saving for the Italian NHS^42^. Interestingly, the enhancing crude rates of UCS/OCS over time recently reported for FVG^16^ might be explained not only by the increasing age of parturients over the years, but also by the augmenting number of TOLAC undertaken. Unfortunately, as explained above, we did not have information on TOLAC attempted, but only on crude rates of VBAC succesfully accomplished, which were clearly increasing from 2005 to 2015 (Figure 3).

PCS and PPCS are not mutually exclusive events against VBAC-1. However, for some maternal or child’s factors with higher risk of PCS, and even more PPCS, the corresponding adjusted probability of VBAC-1 was lower in the present study. This occurred with breech presentation, placentas weighing 600–999 g or 1,000–1,500 g, oligohydramnios, pre-delivery LoS ≥ 3 days and ≥4 US scans during pregnancy, conditions suggesting the role of maternal request and obstetrician’s preference in decision making on DM. Conversely, with small placentas (<500 g), gestations ≥ 41 weeks and especially labour analgesia (strong assocation), the risk of PPCS was very low and VBAC-1’s became high. Labour analgesia is used for pain relief at low concentration. Epidural analgesia prolongs second stage labour, thus increasing the risk of IVD as compared to CS^14,74^.

### Hospital variability by delivery mode

The crude and adjusted rates of all three examined DM (PCS, PPCS, VBAC-1) varied extensively by hospital. The crude rate of PCS ranged from 12.6% (centre G) up to 28.1% (centre B) and the crude rates for PPCS varied from 4.7% (centre G) up to 13.1% (centre B). The adjusted risk of PCS and PPCS were higher in all centres as compared to the reference (centre H), with the only exception of hospital G for PPCS.

The crude rates of VBAC-1 ranged from 12.9% (centre D) up to 46.5% (centre H). At multivariable analysis all hospitals but G were less likely to deliver by VBAC-1 than the reference (centre H), particularly centres B, D, E, F and J. Variation in the VBAC rate is reported in Italian hospitals (ranging from 0% to 25%)^42^ and in other countries, and may be the result of a number of external influences such as economic interests, organizational aspects, medico-legal issues and obstetrician/patient preferences^16,24,75–77^. The adjusted rates of PCS were highest in hospital B and E, characterised also by the lowest adjusted rates of VBAC-1. It can be reasonably argued that fear of medico-legal issues may have been among the main contributors for these figures in latter 2 maternity units^57,76,77^.

However, differences by DM seem to be influenced above all by practice pattern^16^. An Australian study in New South Wales reported a 82% rate of PRCS among 61,894 women with history of one previous CS, with this rate being predominantly attributable to practice pattern (31%) rather than women’s characteristics (17%)^78^. These differences likely reflect in turn variable adherence to standardized obstetric protocols^79^. In a survey on 225 California’s hospitals, 167 of them (74%) allowed VBAC but little adherence to ACOG guidelines was found. The highest compliance with ACOG recommendations regarded procedure and staff resources, whereas guidelines on assessment of patient clinical conditions and eligibility criteria for TOLAC were disregarded^79^. Assessment of patient’s eligibility for TOLAC, fear of medico-legal issues and logistic barriers (distance from centres offering TOLAC) are obstacles to VBAC^80^.

### Prospects

The results of the present study clearly call for a standardization of obstetric practices across FVG maternity centres, with the view of further reducing the number of unnecessary CS. Considering 39.5% (=7,736/19,565) PCS in FVG during 2005–2015 were PPCS, it can be reasonably argued that a number of these planned obstetric procedures may have been uneeded. A series of health policies could be introduced in FVG to target unnecessary CS, further reducing the PCS rate and enhancing the VBAC rate, nearing pictures achieved by the Nordic countries^42^.

PCS and RCS for term singleton pregnancies with cephalic presentation should all be scrutinized^25,81^. Decision-making on PCS should be carefully evaluated (also by second opinion) particularly for breech presentation and those grey areas potentially affected by subjective clinical evaluation, such as higher maternal age, non-reassuring fetal status, obstructed labour, multiple births and isolated oligohydramnios^14^.

There is evidence that successful actions to enhance the VBAC rate include hospital-level interventions (education of staff/patients by opinion leaders, adoption of second opinions for all CS, staffing hospitals with laborists) and provider-level interventions (midwife-led prenatal care, involvement of primary care physicians in childbirth, night float systems for on call staff). By contrast, system-level interventions (education/training of staff and clinical audits), patient-level interventions (prenatal counselling to parturients) and provider guidelines/information achieved mixed outcomes^82^.

A survey on 44 doctors from countries with VBAC rate > 45% outlined that their confidence with VBAC, working in a united obstetric team positively interacting with each other and with women on defined targets (discussed with the parturients) and following structured strategies contributed to enhanced the VBAC rate^83^. This was confirmed by another survey on 71 doctors from countries with VBAC rates < 36%, stressing the importance of a shared informed decision between women and obstetricians, supported by inter-personal trustfulness, adequate clinical skill/confidence of the obstetric team and clinical decisions sustained by scientific evidence^84^. In another qualitative study based upon focus groups on 22 and 51 women from countries with high^85^ and low^86^ VBAC rates, women expressed desire for correct information on VBAC from doctors and to share with them the decision-making on DM. In the latter study interviewees considered VBAC as the first choice in absence of complications and for labour they expected to be assisted by a confident and serene obstetric team, encouraging and supporting TOLAC^85,86^. RCTs conducted in Italy, Germany and Ireland on 2,002 women from 15 different maternity centres, evaluating interventions entailing the use of opinion leaders (one midwife and one obstetrician per maternity centre), education and support to women as well as health care personnel and discussions between operators and women to reach a shared decision on DM showed an enhancement of the VBAC rate from 8% to 22%^87^.

To reach a shared decision on DM women should be informed that those delivering vaginally are generally more satisfied with their own experience as compared to those undergoing CS, even if a planned CS was performed on maternal request^88–90^. These negative feelings in the new mothers may persist up to 10 years following a CS and may cause mood post-partum disorder, interfering also with their parental attitude^91^.

Reaching a shared decision on DM also requires obstetricians as well as women to be informed that a planned TOLAC on term singleton pregnancies with cephalic presentation reportedly has a VBAC success rate of 70–87%^92–94^. Moreover, obstetricians and parturients should also be advised that a history of one previous VD, particularly prior VBAC, is independently and strongly associated with a TOLAC success rate of 85–90%, and with a reduced risk of uterine rupture among women undergoing TOLAC^95^. In addition to using the Robson classification system for comparing the CS rates, it is therefore also important to extrapolate an eventual history of VD and/or VBAC when assessing the success rate of TOLAC^21–34^.

Since PRCS is significantly associated with various obstetric complications (especially hysterectomy and placenta previa/placenta accreta), women desiring multiple pregnancies (≥3) for the future should be fully advised on the advantages of VBAC^95–100^.

Women requesting a TOLAC after 2 previous CS should be counselled of a VBAC successful rate of 71.1% (similar to a history’s of one previous CS), a low risk of uterine rupture (1.36%) and a maternal morbidity comparable to RCS’s^94,101–104^. There is also evidence that the risk of uterine rupture with TOLAC after ≥ 2 previous CS does not differ from a history’s of one single previous CS^105^. Although it is recommended to avoid TOLAC in women with a uterine scar and a history of 3 or more CS^52^, in a USA study of the Consortium of Safe Labour during 2002–2008, 28.8% women with uterine scar out of 228,668 deliveries underwent TOLAC, with a 57.1% VBAC successful rate^24^.

However, a shared decision on DM also implies full information to the woman not only on the benefits of VBAC, but also on the potential serious health risks of a failed TOLAC, which include UCS, intrinsically associated with higher risk of mortality/morbidity for both the woman and the child^32,82^. Women undergoing TOLAC need to be closely monitored post-partum for the risk of uterine rupture, bleeding, and endometritis^106^. The risk of hysterectomy (0.56% vs. 0.19%,) and transfusion (1.99% vs. 1.21%) following TOLAC slightly increases with a history of two previous CS as compared with one^94^. Women should also be counselled that maternal mortality associated with a planned CS in some circumstances may be lower than with a VD^51^. Moreover, women should be informed that a PPCS can offer protection to the pelvic floor after delivery, reducing the risk of incontinence and organ prolapse, which require surgical interventions in 11.1% women during their lifetime^107^. Finally, a planned CS at 39 weeks gestation in some conditions may lower a number of untoward infant outcomes^108,109^.

## Conclusions 

The PCS rate in FVG during 2005–2015 was 19.6%, well below the corresponding national picture of 29% reported for 2010 and slightly under the most recent PCS rate reported for 2017 for the entire Italy (22.2%). By contrast, the VBAC-1 rate was 28.4% (25.4% overall VBAC rate, considering VD on women with at least 1 previous CS), roughly three times the most recent corresponding national pictures (9% in 2017^43^ and 11.4% in 2010^42^) and rather near the 30% VBAC rate recommended by Europeristat^42^. The difference between OCS rate in FVG during 2005–2015 (24.2%) as compared to recent reports from the entire Italy (38.1%) are therefore mainly due to RCS. This confirms the critical role of VBAC in the control of unnecessary CS.

Albeit we observed a marginal decrease in the crude rates of PCS and PPCS over the years in the entire FVG region, accompanied by a progressive enhancement of the VBAC-1 rates over time, endeavours should be made to further reduce the number of uneeded PCS and PPCS and increase the VBAC uptake (especially VBAC-2, on women with 2 previous CS), bringing the OCS rate under 20%, near cut-offs recommended by WHO and figures achieved by the Nordic countries^42,110,111^. We found remarkable variability of DM across FVG hospitals, which likely reflects variable practice pattern sustained by lack of shared obstetric protocols and/or scarce adherence to clinical guidelines. Standardized obstetric protocols should be introduced and enforced at the hospital level to contain the number of redundant PCS, promoting TOLAC where appropriate. PCS and RCS for term singleton pregancies with cefalic presentation should all be audited (also by second opinion)^25^. Decision-making on PCS should be carefully evaluated for breech presentation and those grey areas potentially affected by subjective clinical evaluation, such as higher maternal age, non-reassuring fetal status, obstructed labour, multiple births and isolated oligohydramnios.

All eligible women delivering in FVG hospitals should be offered the option of a TOLAC as a standard policy, especially in centers equipped with an anesthesiology unit dedicated to the labour ward, a blood bank and an interventional radiology unit^95^. These supports allow clinicians to appropriately manage the dramatic, although rare, emergencies associated with a failed TOLAC.

A number of interventions may be adopted to facilitate this process of change: education of staff/patients by opinion leaders; introduction of a second opinion for all CS; prenatal counselling for women and partners^82–85,112^. Although, clinical audits, financial penalties and rewards to maternity centres could also be considered.

Data collected by CEDAP questionnaire in future should be improved to distinguish planned CS and CS performed for failed induction. In order to better control the CS risk, in future the CEDAP questionnaire should also collect information on whether an external version for breech presentation was offered, pursued and the relative outcome (VD, PCS or even UCS). Moreover, the CEDAP questionnare should also collect information on maternal requests for CS (including on eventual tocophobia and/or previous abdominal surgery), TOLAC offered, TOLAC attempted and subsequent outomce (successful VBAC or UCS). 

### Position statement

This work reports the scientific interpretation of health data of FVG made by the authors, it should not be considered an official position of the regional government of FVG.

## Supplementary information

Supplementary File.

## Data Availability

This study analyzed third party data, extracted from the Regional Repository of Friuli Venezia Giulia (FVG), a database anonymously storing potentially sensitive information. Access to this database is therefore subject to permission from the Regional Health Authority of FVG. Contact: Epidemiology & Health Information Service; Central Health Directorate; Health & Social Integration; Social & Family Policies; Via Pozzuolo 330, 33100, Udine, Italy. Tel: + 39 0432 805661; email: salute@certregione.fvg.it.
