# Publisher Correction: Understanding Factors Leading to Primary Cesarean Section and Vaginal Birth After Cesarean Delivery in the Friuli-Venezia Giulia Region (North-Eastern Italy), 2005–2015

**DOI:** 10.1038/s41598-021-85475-0

**Published:** 2021-03-17

**Authors:** L. Cegolon, G. Mastrangelo, G. Maso, G. Dal Pozzo, L. Ronfani, A. Cegolon, W. C. Heymann, F. Barbone

**Affiliations:** 1 Institute for Maternal & Child Health, IRCCS “Burlo Garofolo”, Trieste, Italy; 2 Local Health Unit N.2 “Marca Trevigiana”, Public Health Department, Treviso, Italy; 3 Padua University, Department of Cardio-Thoracic & Vascular Sciences, Padua, Italy; 4 Hospital “Villa Salus”, Obstetric & Gynecology Unit, Venice, Italy; 5 University of Macerata, Department of Political Sciences, Comunication and International Relationships, Macerata, Italy; 6 Florida Department of Health, Sarasota County Health Department, Sarasota, Florida USA; 7 Florida State University, College of Medicine, Department of Clinical Sciences, Sarasota, Florida USA

Correction to: *Scientific Reports* 10.1038/s41598-019-57037-y, published online 15 January 2020

The original version of this Article contained several typographical errors. In the Abstract,

“In our study we examined patterns of PCS, pl compared with planned TOLAC anned PCS (PPCS), vaginal birth after 1 previous CS (VBAC-1) and associated factors in Friuli Venezia Giulia (FVG), a region of North-Eastern Italy, collecting data from its 11 maternity centres (coded from A to K) during 2005–2015.”

now reads:

“In our study we examined patterns of PCS, planned PCS (PPCS), vaginal birth after 1 previous CS (VBAC-1) and associated factors in Friuli Venezia Giulia (FVG), a region of North-Eastern Italy, collecting data from its 11 maternity centres (coded from A to K) during 2005–2015.”

In the Methods section under the subheading ‘Ethics Statement’,

“According to the Italian privacy law (Legislative Decree 101/2018, D.Lgs 101/2018) regional NHS data can be used for scientific purposes within the frame of approved studies/protocols.”

now reads:

“According to the Italian privacy law (Legislative Decree 101/2018, D.Lgs 101/2018) regional data from the Italian National Health Service (NHS) can be used for scientific purposes within the frame of approved studies/protocols.”

In the Methods section under the subheading ‘The database’,

- Polyhydramnios: 657.0;
- Oligohydramnios: 658.0;
- Antepartum hemorrhage, abruptio placentae and placenta previa: 641.(0-1-2-3-8-9);
- Obstructed labour (except shoulder girdle dystocia): 660.(0-1-2-3-5-6-7-8-9);
- Non-reassuring fetal status: 656.3;
- Fetal anomalies: 655.9;
- Cord prolapse: 663.0;
- Premature rupture of membranes (PROM): 658.1;
- Eclampsia/pre-eclampsia: 624.(4-5-6-7);
- Rh iso-immunization: 656.1;

now reads:

- Polyhydramnios: 657.0;
- Oligohydramnios: 658.0;
- Antepartum hemorrhage, abruptio placentae and placenta previa: 641.(0-1-2-3-8-9);
- Obstructed labour (except shoulder girdle dystocia): 660.(0-1-2-3-5-6-7-8-9);
- Non-reassuring fetal status: 656.3;
- Cord prolapse: 663.0;
- Premature rupture of membranes (PROM): 658.1;
- Eclampsia/pre-eclampsia: 642.(4-5-6-7);
- Rh iso-immunization: 656.1;

“The 11 regional maternal facility centres were anonymized and coded by alphabetic letter from A to L.”

now reads:

“The 11 regional maternal facility centres were anonymized and coded by alphabetic letter from A to K.”

In the Methods section under the subheading ‘Conceptual framework’,

“5. Obstetric conditions, shown in Table 5: oligohydriamnios, polyhydramnios, eclampsia/pre-eclampsia, placenta previa/abruptio placenta/ante-partum hemorrhage, non-reassuring fetal status, fetal anomalies, cord prolapse, PROM, RH iso-immunization, obstructed labour (except shoulder girdle dystocia), labour analgesia, labour induction, fetal presentation.”

now reads:

“5. Obstetric conditions, shown in Table 5: oligohydriamnios, polyhydramnios, eclampsia/pre-eclampsia, placenta previa/abruptio placenta/ante-partum hemorrhage, non-reassuring fetal status, congenital malformations at birth, cord prolapse, PROM, RH iso-immunization, obstructed labour (except shoulder girdle dystocia), labour analgesia, labour induction, fetal presentation.”

In the Discussion section under the subheading ‘Key findings’,

“By including also VBAC-2 on women with 2 previous CS (N = 38) the overall VBAC rate in the region would slightly reduce to 25.3%, almost three times the corresponding national rate (9%) reported in 2017 for the entire Italian country^43^.”

now reads:

“By including also VBAC-2 on women with 2+ previous CS (N = 38) the overall VBAC rate in the region would slightly reduce to 25.3%, almost three times the corresponding national rate (9%) reported in 2017 for the entire Italian country^43^.”

In the Discussion section under the subheading ‘Strengths and limitations’,

“We consider the VBAC rates on women with history of only one previous CS in the analysis, since the number of VBAC on women with 2 previous CS was negligible (N = 38).”

now reads:

“We consider the VBAC rates on women with history of only one previous CS in the analysis, since the number of VBAC on women with 2+ previous CS was negligible (N = 38).”

In the Discussion section under the subheading ‘PCS and PPCS’,

“Although suspected macrosomia is an increasing indication for PCS, fetal anomalies were not associated with PCS or PPCS in our study and the level of significance of birthweight ≥ 4,000 g and hypertension/diabetes on the PCS risk was relatively lower^28^.”

now reads:

“Although suspected macrosomia is an increasing indication for PCS, congenital malformations at birth were not associated with PCS or PPCS in our study and the level of significance of birthweight ≥ 4,000 g and hypertension/diabetes on the PCS risk was relatively lower^28^.”

Additionally, the original version of this Article contained errors in Table 5. The ‘Missing’ value for the Factor ‘Placenta previa/abruptio placenta/ante-partum hemorrage’ was given incorrectly as “909”, and now reads “751”. For the same Factor, the value for ‘All Births (N)’ in the ‘Yes’ Stratum row was given incorrectly as “1,182”, and now reads “1,282”. The ‘VBAC-1′ value for the Factor ‘Premature rupture of membranes (Missing: 751)’ in the ‘Yes’ Stratum row was given incorrectly as “30.4”, and now reads “30.8”.

Furthermore, there were errors in the Supplementary File. In Supplementary Table 1, the factor, ‘Placenta previa/abruptio placenta/ante-partum hemorrage’ now reads ‘Placenta previa/abruptio placenta/ante-partum hemorrhage: Yes’; the factor ‘Obstructed labour (but shoulder dystocia)’ now reads ‘Obstructed labour (but shoulder dystocia): Yes’; and the factor ‘Eclampsia/pre-eclampsia’ now reads ‘Eclampsia/pre-eclampsia: Yes’.

In the same table, for the factor, ‘Polyhydramnios: Yes’, the value for ‘PCS’ under the ‘Delivery Mode’ was given incorrectly as “3.39 (2.64; 4.34)”, and now reads “3.56 (2.77; 4.58)”. For the factor ‘Mother’s nationality: Non-EU’, the value for ‘PCS’ under the ‘Delivery Mode’ was given as “1.22 (1.14; 1.30)”, and now reads “1.22 (1.13; 1.30)”. For the factor ‘Mother’s education: secondary’, the value for ‘PCS’ under the ‘Delivery Mode’ was given incorrectly as “1.10 (1.16; 1.32)”, and now reads “1.24 (1.16; 1.32)”. For the factor ‘PROM: Yes’, the value for ‘PPCS’ under the ‘Delivery Mode’ was given incorrectly as “0.43 (0.38; 0.49)”, and now reads “0.43 (0.37; 0.49)”.

In the same table, the rows for the factors ‘Mother’s occupation: Blue collar’ and ‘Father’s age: (40–44) years’ were omitted from the table.

In Supplementary Table 2, the upper bound confidence interval for the ‘PCS’ estimate of Hospital ‘E’ was given incorrectly as “2.33 (2.09; 2.60)”, and now reads “2.33 (2.09; 2.61)”. For the same Hospital, the upper bound confidence interval for the ‘PPCS’ estimate was given incorrectly as “4.75 (3.99; 5.65)”, and now reads “4.75 (3.99; 5.64)”. The upper bound confidence interval for the ‘PCS’ estimate of Hospital ‘G’ was given incorrectly as “0.70 (0.63; 0.89)”, and now reads “0.70 (0.63; 0.79)”. The upper bound confidence interval for the ‘VBAC-1’ estimate of Hospital ‘I’ was given incorrectly as “0.36 (0.27; 0.49)”, and now reads “0.36 (0.27; 0.48)”. The upper bound confidence interval for the ‘VBAC-1′ estimate of Hospital ‘J’ was given incorrectly as “0.41 (0.33; 0.51)”, and now reads “0.41 (0.33; 0.52)”.

Lastly, in the Supplementary File, under the section ‘Results of Supplementary Tables 1 and 2 belong to the same multivariable logistic regression models, adjusted for the same following factors’, the factors ‘maternal age’ and ‘eclampsia/pre-eclampsia’ were omitted from the list under ‘VBAC-1’.

These errors have now been corrected in the HTML and PDF versions of the Article, and in the accompanying Supplementary File.

