# Supplementary material for: Understanding Factors Leading to Primary Cesarean Section and Vaginal Birth After Cesarean Delivery in the Friuli-Venezia Giulia Region (North-Eastern Italy), 2005–2015
Source: Sci Rep. 2020 Jan 15;10:380. doi: 10.1038/s41598-019-57037-y (PMC6962159; doi:10.1038/s41598-019-57037-y)
Supplement: Supplementary file 1 — Supplementary File. [file 41598_2019_57037_MOESM1_ESM.pdf]

Supplementary Table 1. Results of three models of multivariable logistic regression fitted for each delivery mode (Primary Caesarean Section, PCS; Planned PCS, PPCS; Vaginal Birth After 1 previous Caesarean Section, VBAC-1). Significant factors with odds ratio (OR); 95% confidence interval (in brackets) and Benjamini-Hochberg (BH) p-value (bottom of each cell) estimated at 5% false discovery rate (FDR). Obs.= complete (case analysis) observations.

**FACTORS**	**Reference category**	**DELIVERY MODE**		
		**PCS** (95,689 obs.)	**PPCS** (84,764 obs.)	**VBAC-1** (7,942 obs.)
Presentation: breech	Cephalic	108.33 (95.94; 122.33)<br>0	195.88 (1.70.11; 225.56)<br>0	0.19 (0.13; 0.28)<br>1.39E-16
Non reassuring foetal status: Yes	No	13.34 (12.11; 14.70)<br>0	2.80 (2.27; 3.46)<br>2.09E-21	NS
Obstructed labour (but shoulder dystocia)	No	10.20 (9.36; 11.12)<br>0	2.62 (2.18; 3.14)<br>9.36E-25	NS
Placenta previa/abruptio placenta/ante-partum haemorrhage	No	41.11 (33.80; 50.01)<br>0	21.69 (16.97; 27.72)<br>4.80E-132	0.19 (0.08; 0.33)<br>1.56E-06
N. previous livebirths: 1	0	0.39 (0.37; 0.41)<br>2.84E-274	0.46 (0.43; 0.50)<br>1.31E-85	NS
N. previous livebirths: 2	0	0.29 (0.26; 0.32)<br>7.10E-122	0.32 (0.27; 0.37)<br>2.59E-50	NS
Multiple birth	Singleton	8.88 (7.35; 10.73)<br>5.07E-112	9.04 (7.18; 11.39)<br>5.18E-77	NS
Eclampsia/pre-eclampsia	No	5.50 (4.70; 6.44)<br>9.76E-99	2.87 (2.24; 3.68)<br>2.51E-16	0.39 (0.19; 0.82)<br>0.0228
Mother's age: (40-44) years	20-24	3.65 (3.21; 4.15)<br>2.06E-86	4.34 (3.53; 5.32)<br>5.76E-44	NS
Placenta weight: (600-999) g	500-599	1.59 (1.52; 1.68)<br>1.16E-72	1.70 (1.57; 1.83)<br>1.55E-39	0.55 (0.49; 0.62)<br>3.59E-20
Oligohydramnios: Yes	No	2.58 (2.32; 2.87)<br>2.66E-66	2.16 (1.83; 2.56)<br>8.40E-19	0.24 (0.14; 0.43)<br>2.67E-06
Pre-delivery LoS: (3-5) days	<3 days	2.26 (2.05; 2.50)<br>1.15E-56	3.53 (3.07; 4.06)<br>5.35E-69	0.35 (0.23; 0.55)<br>1.03E-05
Mother's age: (35-39) years	20-24	2.32 (2.08; 2.57)<br>8.08E-57	2.46 (2.07; 2.94)<br>3.15E-23	NS
Placenta weight: (1,000-1,500) g	500-599	5.44 (4.38; 6.74)<br>1.37E-53	6.97 (5.32; 9.14)<br>3.60E-44	0.06 (0.02; 0.16)<br>1.13E-07
Birthweight < 2,000g	2,500-4,000	5.03 (4.09;6.19)<br>2.31E-52	2.92 (2.14; 3.99)<br>3.40E-11	NS
Mother's age: 45+ years	20-24	8.64 (6.24; 11.96)<br>4.73E-38	4.34 (3.53; 5.32)<br>5.85E-29	NS
N. previous livebirths: 3	0	0.25 (0.20; 0.31)<br>4.48E-36	0.27 (0.20; 0.37)<br>2.13E-16	NS
Pre delivery LoS: 6+ days	<3	2.49 (2.13; 2.92)<br>2.98E-29	5.01 (4.10; 6.11)<br>5.04E-56	0.34 (0.18;0.64)<br>0.0016
Mother's age: (30-34) years	20-24	1.77 (1.60; 1.95)<br>1.10E-29	1.91 (1.63; 2.25)<br>8.94E-15	NS
Birthweight: (2,000-2,500) g	2,500-4,000	1.85 (1.66; 2.06)<br>1.91E-27	1.59 (1.35; 1.88)<br>6.25E-08	0.55 (0.39; 0.79)<br>0.0018
Polyhydramnios: Yes	No	3.56 (2.77; 4.58)<br>1.36E-22	2.84 (1.94; 4.16)<br>1.50E-07	NS
Labour analgesia: administered	No	0.76 (0.72; 0.80)<br>3.28E-22	0.19 (0.16; 0.21)<br>7.20E-133	4.76 (3.90; 5.81)<br>3.90E-51
N. previous liveborn: 4	0	0.17 (0.12; 0.25)<br>8.37E-22	0.16 (0.10; 0.28)<br>2.26E-12	NS
Cord prolapse: Yes	No	126.68 (44.04; 364.41)<br>6.61E-19	NS	NS
N. US scans during pregnancy: 6+	<4	1.37 (1.28; 1.47)<br>1.02E-18	1.96 (1.75; 2.21)<br>1.78E-29	0.68 (0.58; 0.81)<br>1.90E-15
Gestation: (33-36) weeks	37-40	1.53 (1.39; 1.69)<br>1.40E-16	1.43 (1.23; 1.66)<br>7.54E-06	NS
Neonatal status: Stillborn	Liveborn	0.13 (0.08; 0.21)<br>5.10E-16	0.03 (0.01; 0.08)<br>1.42E-12	NS
Mother's occupation: employed (clerk)	Unemployed/student/housewife	0.79 (0.74; 0.84)<br>7.52E-13	0.77 (0.69; 0.85)<br>3.06E-07	NS
Mother's age: (25-29) years	20-24	1.36 (1.24; 1.49)<br>1.93E-10	1.45 (1.24; 1.69)<br>5.09E-06	NS
Mother's education: junior secondary	University/more	1.24 (1.16; 1.32)<br>9.27E-10	1.19 (1.07; 1.32)<br>0.0013	NS
Gestation: <29 weeks	37-40	0.32 (0.23; 0.46)<br>1.32E-09	0.05 (0.02; 0.09)<br>1.81E-18	NS
N. US scans during pregnancy: 4-5	<4	1.21 (1.13; 1.29)<br>1.76E-08	1.49 (1.33; 1.66)<br>5.72E-12	0.78 (0.67; 0.91)<br>0.0029
Mother's nationality: Non-EU	Italian	1.22 (1.13; 1.30)<br>5.71E-08	NS	NS
Mother's education: primary/none	University/more	1.56 (1.32; 1.84)<br>3.55E-07	1.54 (1.20; 1.97)<br>0.0012	NS
Gestation: 41+ weeks	37-40	1.15 (1.09; 1.21)<br>7.10E-07	0.67 (0.61; 0.74)<br>2.57E-15	1.33 (1.12; 1.57)<br>0.0018
Birthweight: 4,000+ g	2,500-4,000	1.23 (1.13; 1.33)<br>1.03E-06	1.18 (1.04; 1.34)<br>0.0124	NS
Hypertension/diabetes: Yes	No	1.37 (1.20; 1.56)<br>3.86E-06	1.23 (1.01; 1.49)<br>0.0452	NS
Rh iso-immunization: Yes	No	3.27 (1.96; 5.45)<br>3.86E-06	3.63 (1.92; 6.86)<br>0.0001	NS
Placental weight: < 500g	500-599	0.88 (0.83; 0.94)<br>0.0001	0.84 (0.76; 0.92)<br>0.0007	1.36 (1.16; 1.61)<br>0.0004
N. obstetric checks in pregnancy: 8+	4-7	1.11 (1.05; 1.18)<br>0.0002	1.14 (1.05; 1.25)<br>0.0045	NS

Mother's education: secondary	University/more	1.24 (1.16; 1.32)<br>0.0004	NS	NS
Mother's occupation: employed (other)	Unemployed/student/housewife	0.89 (0.83; 0.95)<br>0.0009	0.86 (0.77; 0.95)<br>0.0065	NS
N. previous stillbirths: 1+	0	1.45 (1.12; 1.88)<br>0.0070	1.65 (1.18; 2.31)<br>0.0051	NS
Gestation: (29-32) weeks	37-40	1.45 (1.11; 1.91)<br>0.0088	NS	NS
N. intentional abortions: 2+	0	1.25 (1.05; 1.49)<br>0.0168	NS	NS
Mother's occupation:<br>self-employed/entrepreneurs	Unemployed/student/housewife	0.90 (0.83; 0.98)<br>0.0216	NS	NS
N. obstetric checks in pregnancy: <4	4-7	1.07 (1.01; 1.13)<br>0.0286	1.16 (1.06; 1.27)<br>0.0013	NS
Any Medical Assisted Fertilization: Yes	No	1.28 (1.03; 1.61)<br>0.0355	1.68 (1.25; 2.26)<br>0.0008	NS
Father's age: 55+ years	30-34	1.32 (1.02; 1.70)<br>0.0409	1.98 (1.41; 2.80)<br>0.0002	NS
Mother's nationality: EU (non-Italian)	Italian	0.90 (0.81; 0.99)<br>0.0438	0.83 (0.70; 0.97)<br>0.0261	NS
Mother's occupation:<br>Blue collar	Unemployed/student/housewife	0.93 (0.86; 1.00)<br>0.0477	NS	NS
PROM: Yes	No	NS	0.43 (0.37; 0.49)<br>4.99E-36	NS
Calendar year: (2005-2015)	Linear trend	NS	0.97 (0.95; 0.98)<br>8.37E-09	NS
Father's age: (50-54) years	30-34	NS	1.41 (1.09; 1.82)<br>0.0124	NS
N. previous neonatal deaths: 1+	0	NS	2.14 (1.19; 3.84)<br>0.0144	NS
Father's age: (20-24) years	30-34	NS	0.71 (0.54; 0.93)<br>0.0167	NS
Father's age: (45-49) years	30-34	NS	1.21 (1.03; 1.42)<br>0.0215	NS
Father's age: (40-44) years	30-34	NS	1.12 (1.00; 1.25)<br>0.0492	NS

Supplementary Table 2. Results of three models of multivariable logistic regression for each delivery mode (Primary Caesarean Section, PCS; Planned Primary Cesarean Section, PPCS; Vaginal Birth After 1 previous Caesarean Section, VBAC-1). Hospital estimates (odds ratio, OR) with 95% confidence interval (in brackets) and Benjamini-Hochberg (BH) p-value (bottom of each cell), estimated at 5% false discovery rate (FDR). NS= non-significant. Obs.= complete (case analysis) observations.

Hospital	PCS (95,689 obs.)	PPCS (84,764 obs.)	VBAC-1 (7,942 obs.)
A	1.42 (1.30; 1.56)<br>1.05E-13	2.04 (1.75; 2.39)<br>7.83E-19	0.60 (0.49; 0.75)<br>1.04E-05
B	2.59 (2.37; 2.84)<br>1.18E-95	4.26 (3.66; 4.96)<br>2.64E-76	0.29 (0.23; 0.35)<br>7.54E-30
C	1.15 (1.04; 1.29)<br>0.0113	1.95 (1.64; 2.32)<br>5.46E-14	0.63 (0.49; 0.80)<br>0.0004
D	2.09 (1.83; 2.40)<br>5.33E-26	2.38 (1.90; 2.98)<br>1.16E-13	0.14 (0.09; 0.21)<br>1.76E-20
E	2.33 (2.09; 2.61)<br>2.80E-50	4.75 (3.99; 5.64)<br>6.48E-69	0.16 (0.11; 0.21)<br>2.14E-30
F	1.43 (1.28; 1.61)<br>1.81E-09	1.99 (1.65; 2.40)<br>2.18E-12	0.21 (0.15; 0.30)<br>4.68E-19
G	0.70 (0.63; 0.79)<br>3.28E-09	NS	NS
H	reference	reference	reference
I	1.91 (1.70; 2.14)<br>6.78E-28	2.91 (2.41; 3.52)<br>8.00E-28	0.36 (0.27; 0.48)<br>1.82E-11
J	1.61 (1.46; 1.78)<br>6.31E-22	2.50 (2.13; 2.94)<br>2.84E-28	0.41 (0.33; 0.52)<br>1.51E-14
K	1.33 (1.19; 1.49)<br>5.94E-07	2.61 (2.19; 3.12)<br>5.31E-26	0.47 (0.37; 0.60)<br>3.20E-09

Results of Supplementary Tables 1 and 2 belong to the same multivariable logistic regression models, adjusted for the same following factors:

<u>PCS:</u>

- **Health care setting and timeframe:** hospital;
- **Maternal health factors**: maternal age; hypertension/diabetes; number of US scans in pregnancy; number of obstetric checks in pregnancy; pre-delivery LoS; neonatal status; any medical assisted fertilization;
- **Child's size factors**: gestational age; birthweight; placental weight;
- **Child's fragility factors:** multiple birth;
- **Obstetric history factors:** number of previous livebirths; history of intentional abortions; stillbirth history;
- **Socio-demographic factors**: paternal age; mother's nationality; mother's educational level; mother's occupation;
- **Obstetric factors:** eclampsia/pre-eclampsia; oligohydramnios; polyhydramnios; placenta previa/abruptio placenta/ ante-partum haemorrhage; non-reassuring fetal status; presentation; labour analgesia; obstructed labour (but shoulder girdle dystocia); Rh iso-immunization; cord prolapse.

<u>PPCS:</u>

- **Health care setting and timeframe:** hospital; calendar year;
- **Maternal health factors**: maternal age; hypertension/diabetes; number of US scans in pregnancy; amniocentesis; number of obstetric checks in pregnancy; pre-delivery LoS; neonatal status; any assisted medical fertilization;
- **Child's size factors:** gestational age; birthweight; placental weight;
- **Child's fragility factors:** multiple birth;
- **Obstetric history factors:** number of previous livebirths; number of previous stillbirths; number of previous neonatal deaths;
- **Socio-demographic factors:** paternal age; mother's nationality; mother's educational level; mother's occupation;
- **Obstetric factors**: eclampsia/pre-eclampsia; oligohydramnios; polyhydramnios; placenta previa/abruptio placenta/ ante-partum hemorrhage; non-reassuring fetal status; Rh iso-

immunization; presentation; labour analgesia; obstructed labour (but shoulder girdle dystocia); PROM.

VBAC-1:

- **Health care setting and timeframe:** hospital;
- **Maternal health factors**: maternal age; hypertension/diabetes; number of obstetric checks in pregnancy; number of US scans in pregnancy; pre-delivery LoS;
- **Child's size factors**: gestational age; birthweight; placental weight;
- **Child's fragility factors:** NA;
- **Obstetric history factors:** NA;
- **Socio-demographic factors:** NA;
- **Obstetric factors**: eclampsia/pre-eclampsia; oligo-hydramnios; non-reassuring fetal status; presentation; labour analgesia; placenta previa/abruptio placenta/ ante-partum haemorrhage.
